# The missing link in connective tissue disease care: addressing the shortage of specialist nurses in the UK

**DOI:** 10.1093/rap/rkag031

**Published:** 2026-03-18

**Authors:** Diana Finney, Louise Parker, Will Gregory, Angie Turner

**Affiliations:** Rheumatology, Sussex MSK Health, Sussex, UK; Independent Nurse Consultant, Founder and Director, Raynaud’s Clinic, Louise Parker Associates, London, UK; Rheumatology Department, Salford Royal Hospital, Northern Care Alliance NHS Foundation Trust, Salford, UK; Faculty of Health and Education, Manchester Metropolitan University, Manchester, UK; Philanthropy, The Children’s Trust, Tadworth, UK

CTDs, such as SLE, SSc, idiopathic inflammatory myopathies and overlap syndromes, are among the most complex, high-risk and resource-intensive conditions in rheumatology. SSc remains the rheumatic disease with the highest morbidity and mortality, despite improved survival [1]. Despite advances in pharmacological therapies, a critical workforce gap persists in the UK: the shortage of specialist nurses dedicated to CTD care, which limits the translation of medical advances into improved outcomes.

While specialist nurses are integral to managing RA and SpA, where nurse-led models are embedded within national standards of care, CTD patients often lack disease-specific nursing support. The 2022 British Society for Rheumatology (BSR) State of Play report found that only 7% of rheumatology nurses had a CTD focus, and many departments reported no nurse-led CTD clinics [2]. This stark contrast between gold-standard RA care and CTD provision highlights a persistent inequity, particularly given the complexity and risk profile of CTDs.

CTD patients are at high risk of morbidity and mortality; lupus has a standardised mortality ratio of 2–5 [3] and SSc has a 10-year survival of 66% [4]. They require prompt symptom triage, urgent treatments, regular monitoring, medication counselling and psychosocial support—roles well suited to specialist CTD nurses. A recent systematic review of nurse-led interventions in systemic autoimmune rheumatic diseases demonstrated emerging evidence of improved patient outcomes, care coordination and patient experience, underscoring the central role of nurses in this population [5]. Without CTD specialist nurses, consultants and general practitioners, often undertrained in CTDs, shoulder these responsibilities, causing delays, fragmented care and reactive rather than proactive management.

Lupus data illustrate this inequity further. In the UK, the average time to lupus diagnosis is ≈7.5 years, with some patients reporting delays of several decades, leading to preventable organ damage, psychological distress and reduced trust in healthcare services [6]. Lupus UK (2024) highlights the particular value of specialist lupus nurses, particularly for patients from ethnic minority backgrounds who often face greater severity and barriers to timely diagnosis [3].

SSc highlights structural health inequalities. It affects far more women than men, often during working age, and is linked to high work disability and early exit from employment, with significant socio-economic impacts. This aligns with government priorities to support people to stay in or return to work. Ethnic minority patients experience poorer outcomes and greater barriers to specialist care, underscoring the need for proactive, expert nursing support.

Specialist CTD nurses are vital for coordinating multidisciplinary care, including respiratory physicians for interstitial lung disease, nephrologists for lupus nephritis, dermatologists for refractory skin disease, obstetricians for high-risk pregnancies, gastroenterologists and nutrition teams and allied health professionals such as physiotherapists, occupational therapists and hand therapists. Without a designated nurse, patients frequently fall through the gaps, particularly during transitions between primary and secondary care or during acute deterioration. Visible nurse presence on ward rounds and clinics improves continuity, safety and patient confidence, providing clear access points via advice lines and rapid review pathways.

The National Health Service (NHS) Long Term Plan explicitly prioritises workforce retention, specialist skill development and a reduction of unwarranted variation in care, yet CTD nursing remains underdeveloped nationally. A 2021 NHS benchmarking audit found only 18% of Trusts had a named CTD nurse role [7]. Most CTD patients are seen by generalist nurses whose caseloads are dominated by rheumatoid arthritis and biologic monitoring, leaving little room for proactive CTD management. This lack of succession planning threatens retention of specialist knowledge, particularly as experienced nurses retire early—directly contradicting NHS goals to retain senior staff and preserve expertise.

The 2025 Royal College of Nursing Rheumatology Nurse Competency Framework [6] acknowledges the need for disease-specific expertise and career pathways in CTDs, but progress remains inconsistent [8]. Investment in CTD nursing therefore aligns directly with NHS Long Term Plan commitments to workforce sustainability, patient-centred care and prevention of avoidable hospital admissions.

Investing in CTD specialist nurses is both good practice and good policy. Established models, such as nurse-led lupus clinics at Guy’s and St Thomas’ NHS Foundation Trust and the systemic sclerosis service at Royal Free Hospital NHS Foundation Trust, demonstrate improved patient satisfaction, reduced flares and lower unplanned admissions through timely interventions and follow-up. These service models support the objectives of the Department of Health and Social Care Rare Diseases Action Plan 2024, emphasising equitable access to specialist expertise [9]. Replicating such models nationally could reduce crisis-driven care and support regional centres of excellence for CTD nursing development.

In a strained system, the most vulnerable rheumatology patients still face fragmented care. Addressing the shortage of CTD specialist nurses should be a national priority supported by NHS workforce plans that commit to improving outcomes. This requires robust job planning templates, including protected time in a CTD nurse’s role. Current provision is inequitable and unsustainable. The BSR 2023–2026 strategy calls for reducing variation in care, building the multidisciplinary workforce and embedding patient-centred models [10]. CTD nursing expansion directly supports these objectives and should be embedded in service commissioning frameworks.

The broader nursing workforce crisis—high attrition, early retirement and burnout—threatens specialist rheumatology care. CTD nursing roles, if properly resourced, offer meaningful career progression, improved job satisfaction and an opportunity to retain highly skilled nurses while delivering safer, more equitable care for patients.

Given the vulnerability and complexity of CTD patients, they deserve tailored, expert support delivered by nurses with the knowledge, time, authority and clinical confidence to act. The shortage of CTD specialist nurses represents more than a workforce gap, it is a failure of strategic long-term planning that must be urgently addressed nationally.

## Data Availability

No new data were generated or analysed in support of this article.
